# Prevalence and trends of aminoglycoside resistance in *Shigella* worldwide, 1999-2010

**DOI:** 10.7555/JBR.27.20120125

**Published:** 2013-02-28

**Authors:** Bing Gu, Xing Ke, Shiyang Pan, Yan Cao, Ling Zhuang, Rongbin Yu, Huimin Qian, Genyan Liu, Mingqing Tong

**Affiliations:** a Department of Laboratory Medicine, the First Affiliated Hospital, Nanjing Medical University, Nanjing, Jiangsu 210029, China;; b National Key Clinical, Department of Laboratory Medicine, Nanjing, Jiangsu 210029, China;; c Department of Acute Infectious Disease Prevention and Control, Jiangsu Provincial Center for Disease Prevention and Control, Nanjing, Jiangsu 210029, China;; d Department of Epidemiology and Biostatistics, School of Public Health, Nanjing Medical University, Nanjing, Jiangsu 210029, China.

**Keywords:** *Shigella*, aminoglycoside, resistance, patterns, prevalence, trends, meta-analysis

## Abstract

Shigellosis causes diarrheal disease in humans in both developed and developing countries, and multi-drug resistance in *Shigella* is an emerging problem. Understanding changing resistance patterns is important in determining appropriate antibiotic treatments. This meta-analysis systematically evaluated aminoglycoside resistance in *Shigella*. A systematic review was constructed based on MEDLINE and EMBASE databases. Random-effect models or fixed-effect models were used based on *P* value considering the possibility of heterogeneity between studies for meta-analysis. Data manipulation and statistical analyses were performed using software STATA 11.0. By means of meta-analysis, we found a lower resistance to three kinds of aminoglycosides in the Europe-America areas during the 12 year study period than that of the Asia-Africa areas. Kanamycin resistance was observed to be the most common drug resistance among *Shigella* isolates with a prevalence of 6.88% (95%CI: 6.36%-7.43%). Comparison of data from Europe-America and Asia-Africa areas revealed that *Shigella flexneri* resistance was greater than the resistance calculated for *Shigella sonnei*. Importantly, *Shigella sonnei* has played a significant role in aminoglycoside-resistance in recent years. Similarly, data showed that resistance to these drugs in children was higher than the corresponding data of adults. In conclusion, aminoglycoside-resistant *Shigella* is not an unusual phenomenon worldwide. Distribution in *Shigella* resistance differs sharply based on geographic areas, periods of time and subtypes. The results from the present study highlight the need for continuous surveillance of resistance and control of antibiotic usage.

## INTRODUCTION

Acute gastroenteritis and diarrheal diseases continue to be a health problem worldwide, especially in developing countries. They account for approximately 2.5 million deaths per year in children < 5 years of age[1],[2]. Worldwide, the most common bacterial pathogens causing these diseases are: *Salmonella* spp, *Shigella* (*S*.) spp, *Campylobacter* spp, *Escherichia coli* O157:H7, *Listeria monocytogenes*, *Vibrio cholerae*, and *Yersinia enterocolitica*[3],[4]. The common route of infection by these pathogens is the ingestion of contaminated food and drinks[5]. Infection by *Shigella* species is an important global public health problem[6]. *Shigella* infections, especially *S. flexneri* and *S. sonnei* infections, can lead to illness ranging from mild, self-limited diarrhea to severe dysentery with frequent passages of blood and mucus, high fever, cramps, tenesmus, and in rare cases, bacteremia. Complications of shigellosis are seen most frequently in children, the elderly, and the immunocompromised. Therefore, shigellosis is recognized by the World Health Organization (WHO) as major global public health concern[7],[8].

Prompt treatment with effective antimicrobial agents shortens the duration of symptoms and carriage, and reduces the spread of infection. However, antimicrobial resistance has complicated the selection of empirical agents for the treatment of shigellosis, particularly in children. *Shigella* isolates often showed resistance to commonly used, inexpensive antimicrobials, including ampicillin, piperacillin, trimethoprim-sulfamethoxazole, thereby drastically reducing therapeutic possibilities. Thus, the use of sulfonamide or β-lactam antibiotics would not be appropriate for empirical treatment of shigellosis. Shigella strains have become progressively resistant to multiple antimicrobial agents, initially to sulfonamides[9],[10], shortly after they became commercially available; resistance to tetracycline, chloramphenicol, and streptomycin was seen less than 10 years after each was introduced, with subsequent resistance to ampicillin, kanamycin, and trimethoprim-sulfamethoxazole[11],[12]. In certain eastern Africa populations and in a study from China, aminoglycoside resistance of Shigella is a common finding[13],[14].

The present study aimed to identify the worldwide prevalence and distribution of aminoglycoside-resistant *Shigella* using meta-analysis based on data gathered from a systematic review of articles reported between January 1999 and July 2012. The relevant estimates were evaluated for new cases and previously treated cases, respectively, which could provide a clear profile for the status of aminoglycoside-resistant *Shigella* globally.

## MATERIALS AND METHODS

### Literature identification

We conducted a computerized search of MEDLINE (January 1999--July 2012) and EMBASE (January 1999--July 2012) to identify all reports on aminoglycosides resistance associated with *Shigella* infections. The following keywords were used in searches: “bacterial surveillance” or “antimicrobial resistance” or “bacterial resistance” and “*Shigella*”[15]. We also attempted to identify potentially relevant articles by checking the references of the germane articles and through personal communications with colleagues.

### Inclusion and exclusion criteria

Two investigators (BG and XK) reviewed potentially appropriate studies independently, to determine whether they met predetermined eligibility criteria. Disagreements between the reviewers were resolved by consensus. Studies obtained from the literature search were checked by title and citation. If an article appeared relevant, the abstract was reviewed. Relevant abstracts were examined in full text. The inclusion and exclusion criteria were established by the investigators prior to review of the literature. The inclusion criteria were as follows: original article, short communication, correspondence or letter which provided sufficient original data, and all strains isolated from stool. Studies were excluded if they met the following conditions: (1) review or case report; (2) not 1999--2010 data; (3) not separated by country/region; (4) non-human bacterial source; (5) did not include study drugs; (6) did not include resistance results for study pathogens; (7) inclusion/exclusion criteria were not presented; (8) non-recommended regimens/dosing; (9) susceptibility results were not presented. Before we excluded the studies, authors of such studies were contacted in an effort to obtain missing data.

### Validity assessment

Studies were assessed for quality and only high quality studies were included for analysis. Characteristics of high quality studies were: prospective cohort, retrospective consecutive cohort; provided basic data including study period and area, total tested numbers and resistant numbers; susceptibility test was performed in accordance with guidelines established by the Clinical and Laboratory Standard Institute (CLSI)[[16]; reported at least one of three antimicrobials (gentamicin, kanamycin and amikacin) with quality control; individuals included in studies had no infections other than bacillary dysentery. Only one representative case for each outbreak was included, unless the isolates had different antibiotic susceptibility patterns. When study strains overlapped, we included strains from the more recent and larger study in the analysis. If the strains from the smaller study provided data that was not reported in the larger study, results were included for that specific variable.

### Data extraction and statistical analysis

Data extraction was performed by two reviewers (BG and XK) using a standardized extraction form. When there was disagreement, the relevant paper was reviewed and differences were resolved by consensus. Microsoft Excel (version 12.0) software was used for data entry and analysis. In our review, considering the possibility of significant heterogeneity between studies which were tested with the Q test (*P* < 0.10 was considered indicative of statistically significant heterogeneity), random effect models or fix effect models were chosen by *P* value for meta-analysis. Freeman-Tukey arcsin transform to stabilize variances, and after the meta-analysis, investigators can transform the summary estimate and the CI boundaries back to proportions using sin function. Specific conversion details were previously described[17]. Data manipulation and statistical analyses were undertaken using the Statistical Software Package (STATA) 11.0 (STATA Corporation, College Station, TX, USA).

## RESULTS

### Studies and endpoints

**Fig. 1:**
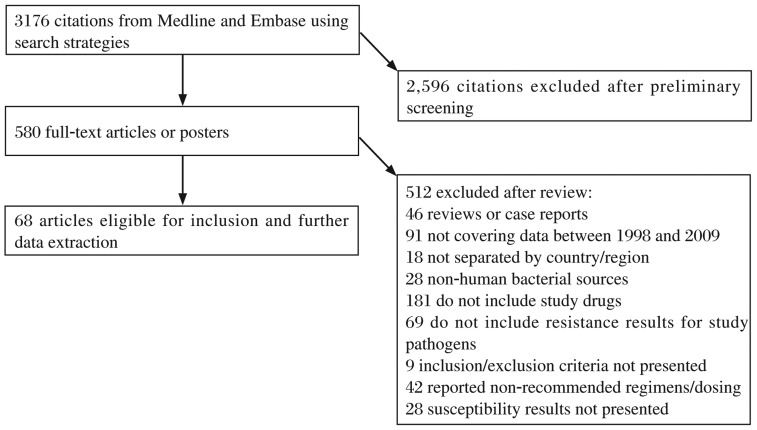
Flow diagram of study identification.

We reviewed 3,176 publications from MEDLINE and EMBASE reported from 1999 to 2012. Candidate articles are shown in ***Fig. 1***. After exclusion based on title and abstract evaluation, 580 articles were retrieved for detailed, full-text evaluation. As shown in ***Fig. 1***, among the included articles, 46 studies were reviews or case reports. Ninety-one articles did not use data that was within the 12-year study period. Findings in 18 articles were not separated by country/region. Human or resistance results for study pathogens were not presented in 28 and 69 studies, respectively. Detailed results of drug susceptibility testing (DST) with respect to study drugs were not provided in 181 studies. Recommend regimens, recommended dosing or data on minimal inhibitory concentrations (MIC) was not included in 9, 42, and 28 studies, respectively. Finally, 68 studies, addressing the prevalence of aminoglycoside-resistant *Shigella* in new cases or in previously treated cases, were identified.

### Status of aminoglycoside-resistant *Shigella*

***Table 1*** shows the meta-analysis of the global status of *Shigella* aminoglycoside resistance in new cases or in previously treated cases worldwide. The summarized prevalence of gentamicin, kanamycin and amikacin resistance was found to be 3.95% (95%CI: 3.59%-4.22%) (n/N= 937/14,059), 6.88% (6.36%-7.43%) (n/N=1,106/8,647) and 1.29% (0.97%-1.68%) (n/N=432/8,614), respectively. Importantly, evident heterogeneity was observed (*P* < 0.001). In the stratified analyses, the prevalence of any drug resistance was observed to vary by geographic areas, study years and subtypes. Lower rates were observed for studies from Europe-America and the period of 1999 to 2004, while the rates from Asia-Africa and using the subtypes of *S. flexneri* were higher. The end time for enrollment of the cases (after 2008) did not significantly change the results.

**Table 1:** Status of aminoglycoside-resistant *Shigella* from 1999-2010

Antimicrobial agents	Classification of drug desistance	Prevalence of drug resistance (95% CI) (%)	n/N	No. of studies
Gentamicin	Total resistance	3.95 (3.59-4.22)	937/1,4059	94
	Stratified by geographic areas			
	America	0.34 (0.19-0.52)	20/6,510	15
	Europe	1.59 (0.50-3.26)	7/480	2
	Africa	8.37 (0.98-21.98)	128/1,093	14
	Asia	10.65 (7.72-14.00)	784/6,064	63
	Stratified by years			
	1999-2001	2.62 (2.09-3.21)	152/3,107	27
	2002-2004	4.74 (4.28-5.20)	592/8,034	44
	2005-2007	5.77 (5.26-6.31)	630/7,371	35
	2008-2010	1.73 (1.26-2.26)	7/1,313	11
	Stratified by subtypes			
	*S. flexneri*	4.64 (3.85-5.47)	190/2,605	46
	*S. sonnei*	0.79 (0.58-1.02)	116/6,448	89
Kanamycin	Total resistance	6.88 (6.36-7.43)	1,106/8,647	30
	Stratified by geographic areas			
	America	0.59 (0.32-0.95)	32/5,592	12
	Europe	0.58 (0.02-1.88)	1/252	1
	Africa	8.65 (7.81-9.32)	67/77	1
	Asia	16.78 (7.58-28.71)	1,007/2,978	16
	Stratified by years			
	1999-2001	3.12 (2.40-3.95)	121/1,928	14
	2002-2004	11.09 (10.29-11.92)	987/5,607	15
	2005-2007	12.05 (11.18-14.21)	960/5,029	9
	2008-2010	0.48 (0.23-0.84)	8/1,922	3
	Stratified by subtypes			
	*S. flexneri*	9.25 (7.69-10.96)	182/1,198	19
	*S. sonnei*	1.06 (0.79-1.35)	170/5,150	25
Amikacin	Total resistance	1.29 (0.97-1.68)	432/8,614	37
	Stratified by geographic areas			
	North America	0.00 (0.00-0.01)	0/4,432	10
	Europe	N/A	N/A0	0
	Africa	10.69 (1.57-34.76)	14/392	6
	Asia	16.29 (13.35-19.67)	421/4,075	25
	Stratified by years			5
	1999-2001	1.01 (0.61-1.54)	48/1,990	14
	2002-2004	5.00 (4.41-5.60)	390/5,136	22
	2005-2007	5.40 (4.76-6.10)	396/4,393	19
	2008-2010	0.70 (0.20-1.53)	24/602	2
	Stratified by subtypes			
	*S. flexneri*	2.62 (1.80-3.59)	52/1203	19
	*S. sonnei*	0.30 (0.16-0.49)	49/4137	17

*n*: number of events(drug resistance); N: total number of patients from the included studies; N/A: results not available.

The most common drug resistance was observed for kanamycin. Among kanamycin resistance, the highest drug resistance rate by geographic areas was found in Asia with a prevalence of 16.78% (7.58%-28.71%). Similarly, the most common resistance was observed for 2005-2007 and *S. flexneri* with a summarized combined prevalence of 12.05% (11.18%-14.21%) and 9.25% (7.69%-10.96%), respectively.

### Studies considered for primary analysis

A total of 68 reports were included in the meta-analysis. As specified a priori in our analysis plan, meta-analyses were performed for outcomes in which there were one or more observations of pediatric or adult group resistance that could be aggregated in the three drugs. Analyses were conducted across the geographic areas, study years and different subtypes for selected primary endpoints, including the resistance of *Shigella* to gentamicin, kanamycin and amikacin; *S. flexneri* to gentamicin, kanamycin and amikacin; *S. sonnei* to gentamicin, kanamycin and amikacin ( ***Supplementary Table 1*** available online).

### Resistance patterns in European-American and Asian-African countries

#### Gentamicin

***Table 2*** shows the resistance rates of total *Shigella* isolates in European-American and Asian-African countries. A lower prevalence of gentamicin resistance was found in European-American countries at 0.68% (0.39%-1.05%). After analyzing the study data on years, we observed a minimal change in the resistance prevalence of gentamicin, from 0.25% (0.04%-0.64%) to 0.84% (0.08%-2.40%) in Europea-American countries, in contrast to data in Asian-African countries, which fluctuated from 6.05% (1.18%-14.28%) to 20.83% (12.67%-30.40%). It is worth noting that the resistance prevalence of gentamicin increased annually in Asian-African countries, while the resistance prevalence decreased year by year in European-American countries.

**Table 2:** Resistance to gentamicin, kanamycin and amikacin in *Shigella* spp. collected during 1999-2010

Antibiotic	Study period	Europe-America			Asia-Africa		
		No. of studies	Resistancet rate%(95%CI)	%Weight	No. of studies	Resistancet rate%(95%CI)	%Weight
Gentamicin	1999-2001	5	0.84 (0.08-2.40)	19.76	22	6.05 (1.18-14.28)	21.84
	2002-2004	9	0.75 (0.19-1.67)	33.58	37	10.17 (6.71-14.21)	37.84
	2005-2007	9	0.72 (0.35-1.23)	33.82	34	12.77 (8.82-17.38)	32.60
	2008-2010	3	0.25 (0.04-0.64)	12.85	8	20.83 (12.67-30.40)	7.71
	Overall	26	0.68 (0.39-1.05)	100.00	101	10.81 (8.34-13.52)	100.00
Kanamycin	1999-2001	4	0.87 (0.45-1.42)	21.26	10	20.96 (3.37-48.11)	44.45
	2002-2004	6	0.64 (0.19-1.33)	32.19	9	14.00 (3.97-28.85)	39.61
	2005-2007	6	0.58 (0.14-1.28)	31.55	3	32.40 (17.87-48.91)	15.94
	2008-2010	2	0.47 (0.20-0.85)	15.00	0	N/A	N/A
	Overall	18	0.60 (0.37-0.88)	100.00	22	19.63 (11.85-28.80)	100.00
Amikacin	1999-2001	4	0.28 (0.00-1.08)	31.69	10	6.39 (1.40-14.63)	21.41
	2002-2004	4	0.25 (0.00-1.01)	33.34	18	9.25 (4.83-14.88)	41.56
	2005-2007	3	0.06 (0.00-0.26)	25.58	16	8.54 (3.70-15.16)	34.77
	2008-2010	1	0.05 (0.04-0.40)	9.38	1	48.06 (34.57-61.65)	2.26
	Overall	12	0.16 (0.03-0.40)	100.000	45	8.90 (6.00-12.34)	100.000

N/A: results not available.

#### Kanamycin

The kanamycin resistance calculations of *Shigella* isolates among different areas are shown in ***Table 2***. The prevalence of gentamicin resistance in Asian-African countries increased sharply from 14.00% (3.97%-28.85%) in 2002-2004, to 20.96% (3.37%-48.11%) in 1999-2001 and to 32.40% (17.87%-48.91%) in 2005-2007. Data for Asian-African regions from 2008-2010 were not found. The changes in kanamycin resistance in European-American countries were minimal; in fact, the resistance prevalence decreased annually.

#### Amikacin

***Table 2*** compares the amikacin resistance of *Shigella* isolates between European-American and Asian-African countries. In European-American regions, a lower amikacin resistance was also found during the 12-year study period. In fact, amikacin resistance decreased from 0.28% (0.00-1.08) to 0.05% (0.04-0.40). The highest resistance of *Shigella* isolates to amikacin was only 0.28% (0.00%-1.08%). We observed that the prevalence of amikacin resistance remarkably increased from 6.39% (1.40%-14.63%) to 48.06% (34.57%-61.65%) in Asian-African countries.

#### Comparison between S. flexneri and S. sonnei

Comparison of the data from Europe-America or Asia-Africa revealed that *S. flexneri* resistance to gentamicin [0.65% (0.30%-1.14%); 9.66% (5.51%-14.81%)] was greater than the resistance calculated for *S. sonnei* [0.39% (0.21%-0.61%); 13.66% (7.72%-20.96%)] (***Tables 3***, ***4***, and ***5***). Similarly, *S. flexneri* resistance to kanamycin [1.59% (0.88%-2.51%); 31.28% (13.97%-51.86%)] was greater than the resistance calculated for *S. sonnei* [0.30% (0.15%-0.51%); 18.10% (4.89%-37.20%)]. *S. flexneri* resistance to amikacin [0.37% (0.05%-0.96%); 7.72% (3.37%-13.66%)] was greater than the resistance calculated for *S. sonnei* [0.07% (0.01%-0.18%); 6.91% (1.75%-15.09%)]. ***Tables 3*** to ***5*** also show that gentamicin and kanamycin were observed for the most common drug resistance system with a summarized prevalence of 33.95% (3.75%-75.11%) and 64.16% (57.04%-70.99%) in Asia-Africa, respectively. In European-American or Asian-African regions, amikacin resistance incidence of *S. flexneri* was not very high at 0.37% (0.05%-0.96%) and 7.72% (3.37%-13.66%), respectively. A difference was found in European-American countries where the prevalence of *S. sonnei* resistance was greater in the period of 2008-2010, giving a 7.18-fold increase in gentamicin-resistance and 4.41-fold increase in kanamycin-resistance from 1999 to 2010. In Asian-African regions, resistance data about *S. flexneri* or *S. sonnei* during 2008-2010 were not found.

**Table 3:** Rates of resistance to gentamicin in *S. flexneri* and *S. sonnei* isolated from Europe-America and Asia-Africa during 1999-2010

Regions	Study period	*S. flexneri*			*S. sonnei*		
		No. of studies	Resistance rate% (95%CI)	% Weight	No. of studies	Resistance rate% (95%CI)	% Weight
Europe-America	1999-2001	3	0.29 (0.00-1.33)	18.29	3	0.33 (0.06-0.82)	13.93
	2002-2004	6	0.85 (0.18-2.03)	31.14	7	0.31 (0.05-0.78)	32.06
	2005-2007	7	0.97 (0.29-2.02)	36.57	8	0.61 (0.20-36.40)	33.06
	2008-2010	2	0.24 (0.05-1.40)	14.00	3	0.28 (0.05-0.70)	17.61
	Overall	18	0.65 (0.30-1.14)	100.00	21	0.39 (0.21-0.61)	100.00
Asia-Africa	1999-2001	11	10.44 (1.00-28.04)	26.10	12	5.49 (0.88-13.69)	32.07
	2002-2004	14	9.05 (2.91-18.14)	36.25	15	8.26 (2.19-17.68)	35.04
	2005-2007	11	8.93 (3.07-17.45)	25.56	10	30.54 (10.41-55.70)	19.94
	2008-2010	5	12.74 (8.45-17.76)	12.08	6	33.95 (3.75-75.11)	12.95
	Overall	18	9.66 (5.51-14.81)	100.00	43	13.66 (7.72-20.96)	100.00

**Table 4:** Rates of resistance to kanamycin reported for *S. flexneri* and *S. sonnei* isolated from Europe-America and Asia-Africa during 1999-2010

Regions	Study period	*S. flexneri*			*S. sonnei*		
		No. of studies	Resistance rate% (95%CI)	% Weight	No. of studies	Resistance rate% (95%CI)	% Weight
Europe-America	1999-2001	3	0.65 (0.04-2.00)	20.31	3	1.13 (0.54-1.93)	17.19
	2002-2004	4	2.60 (0.95-5.04)	30.00	4	0.18 (0.05-0.40)	32.27
	2005-2007	4	1.52 (0.31-3.62)	30.03	3	0.16 (0.01-0.48)	19.81
	2008-2010	2	1.78 (0.41-4.10)	16.62	3	0.25 (0.06-0.57)	30.74
	Overall	13	1.59 (0.88-2.51)	100.00	13	0.30 (0.15-0.51)	100.00
Asia-Africa	1999-2001	5	31.56 (3.01-72.47)	41.44	10	18.80 (3.93-41.30)	56.25
	2002-2004	4	27.90 (2.68-66.07)	37.62	7	11.22 (0.27-45.02)	37.61
	2005-2007	2	35.42 (0.04-90.30)	20.94	1	64.16 (57.04-70.99)	6.13
	2008-2010	0	N/A	N/A	0	N/A	N/A
	Overall	11	31.28 (13.97-51.86)	100.00	18	18.10 (4.89-37.20)	100.00

N/A: results not available.

**Table 5:** Rates of resistance to amikacin reported for *S. flexneri* and *S. sonnei* isolated from Europe-America and Asia-Africa during 1999-2010

Regions	Study period	*S. flexneri*			*S. sonnei*		
		No. of studies	Resistance rate% (95%CI)	% Weight	No. of studies	Resistance rate% (95%CI)	% Weight
Europe-America	1999-2001	3	0.29 (0.00-1.33)	37.54	3	0.08 (0.00-0.38)	24.02
	2002-2004	3	0.40 (0.01-1.80)	27.71	1	0.05 (0.04-0.41)	14.61
	2005-2007	3	0.40 (0.01-1.78)	27.86	5	0.07 (0.00-0.25)	47.80
	2008-2010	1	0.53 (0.49-4.59)	6.89	1	0.05 (0.05-0.44)	13.57
	Overall	10	0.37 (0.05-0.96)	100.00	10	0.07 (0.01-0.18)	100.00
Asia-Africa	1999-2001	5	5.60 (0.76-14.52)	32.54	4	4.81 (0.38-13.72)	32.51
	2002-2004	5	11.85 (5.02-21.08)	38.75	5	7.96 (0.69-22.11)	46.25
	2005-2007	3	6.05 (0.02-21.49)	28.71	2	7.88 (2.81-45.17)	21.24
	2008-2010	13	N/A	N/A	0	N/A	N/A
	Overall	26	7.72 (3.37-13.66)	100.00	11	6.91 (1.75-15.09)	100.00

N/A: results not available.

#### Comparison between children and adults

***Table 6*** shows the clear relationship between the resistance rate and the age of patients with diarrhea. Strains explicitly isolated from children were naturally classified into the pediatric group, whereas the remaining strains were viewed as isolates from adults. For *Shigella*, an additional analysis was conducted for a pediatric group population (34 studies).

**Table 6:** Antimicrobial resistance in children and adults

Group	Gentamicin			Kanamycin			Amikacin		
	No. of studies	Resistance rate% (95%CI)	% Weight	No. of studies	Resistance rate% (95%CI)	% Weight	No.of studies	Resistance rate% (95%CI)	% Weight
Adults	68	5.93 (3.97-8.23)	67.50	28	5.40 (1.87-10.62)	92.84	25	2.23 (0.81-4.35)	61.38
Children	34	18.34 (9.81-28.76)	32.50	2	70.72 (33.95-96.25)	7.16	20	8.43 (3.26-15.71)	38.62
Overall	102	9.51 (6.94-12.44)	100.00	30	8.40 (3.22-15.71)	100.00	45	4.28 (2.25-6.91)	100.00

In this pediatric group, the resistance of *Shigella* to gentamicin was higher than that among the adult group population [5.93% (3.97%-8.23%) and 18.34% (9.81%-28.76%)]. Kanamycin resistance in the pediatric group was significantly higher than that in the adult group, which showed 70.72% (33.95%-96.25%) versus 5.40% (1.87%-10.62%) for kanamycin. Similarly, greater resistance to amikacin was shown in the pediatric group than in the adults group [8.43% (3.26%-15.71%) vs 2.23% (0.81%-4.35%)].

## DISCUSSION

In China, *Shigella* spp. is the most frequently isolated gastrointestinal pathogen and accounts for up to 1.7 million episodes of bacillary dysentery annually, with up to 200,000 patients admitted to hospitals[18]. Any of four subtypes of *Shigella* (*S. dysenteriae, S. flexneri, S. boydii*, and *S. sonnei*) can cause shigellosis. Children are at a higher risk of being affected by the disease, which might be a reflection of secondary infection from the adults as well as poor personal hygiene[19]. Because of increasing antimicrobial resistance to *Shigella*, empiric treatment options are dwindling. In a recent study, aminoglycosides showed higher in vitro activity against members of the family *Enterobacteriaceae*. The function of aminoglycoside antibiotics for empirical treatment of patients with serious infections caused by *Shigella* merits our attention.

As with other classes of antibiotics, significant differences in the spectrum of antimicrobial activity exist among various aminoglycosides[20]. Aminoglycosides bind to the bacterial ribosome and inhibit protein synthesis. Generally, newer aminoglycosides, such as gentamicin, tobramycin, amikacin, netilmicin, isepamicin, dibekacin, and arbekacin, have broader spectra of activity than older compounds like streptomycin and kanamycin. Aminoglycosides are often administered in combination with other antibacterial agents. Despite their potential nephrotoxicity, ototoxicity and problems associated with aminoglycoside-resistant organisms, aminoglycoside antibiotics remain valuable and sometimes indispensable for the treatment of various infections and prophylaxis in special situations[21]. Several mechanisms have been proposed for bacterial resistance to aminoglycoside antibiotics, including decreased antibiotic uptake and accumulation, modification of the ribosomal target, efflux of antibiotic, and enzymatic modification of aminoglycosides.

The resistance of clinical isolates to aminoglycoside antibiotics varies with the specific drug, the microorganism, mechanism of resistance, geographic area, and many other factors. Of the 580 articles and abstracts fully evaluated for this meta-analysis, only 68 published reports met our strict inclusion criteria. Several studies which we reviewed were not included because they did not report on the outcomes of the study drugs in the study period or did not meet other inclusion criteria. However, use of a statistical tool to assess potential heterogeneity among included studies (STATA) gave us further confidence in the meta-analysis results. This meta-analysis demonstrated that the resistance to aminoglycoside is a significant issue in Asia and Africa, as well as in Europe and America. Reported aminoglycoside resistance in *Shigella* varies greatly from country to country and is likely to be an important problem in certain regions. The goals of our study were to analyze the distribution of antimicrobial resistance associated aminoglycoside-resistant *Shigella* based on articles reported between January 1999 and December 2010 and to examine issues related to reasonable treatment about shigellosis.

The first noteworthy finding of this study was that all of these three drugs, in the European-American regions, had an obviously lower resistance rate during the 12 years than in the Asian-African regions. Regardless of its origins and mechanisms, the widespread resistance that we found among *S. flexneri* and *S. sonnei* suggests that infections due to drug-resistant *Shigella* are now endemic around the world. Although mild illness can resolve without antimicrobial treatment, current recommendations guide clinicians to treat *Shigella* infections with antimicrobial therapy to reduce the duration and severity of clinical symptoms and decrease the shedding period of *Shigella*. Because isolation and antimicrobial susceptibility testing for *Shigella* take only several days, antimicrobial agents were generally selected empirically. Combined with the characteristics of antibiotics use in different areas, we found that the use trends of aminoglycoside for shigellosis were not very serious. Selected literature reported from America[22],[23] showed that trimethoprim-sulfamethoxazole and ampicillin can no longer be considered appropriate empirical therapies. A Chinese article found that[18],[24], for the treatment of shigellosis, 35 to 56 different antibiotics were used, with penicillin, cephalosporin, and macrolides accounting for the largest total volume. In some cases, aminoglycoside is effective and approved for use as treatment of *Enterobacteriaceae*. Stronger antibiotics like aminoglycoside may also present notable side effects. Rising resistance rates of these stronger antibiotics have a major impact on the ability of physicians to treat common infections. Patients, especially from Asian-African regions, faced more severe infections with increased duration as resistance increases. They may also experience heightened toxicity associated with the use of stronger antibiotics. Clinicians eventually encountered infections caused by highly resistant pathogens for which no effective antibiotics are available.

To compare the resistance difference among *Shigella* species, additional clinical data from large-scale observational studies were needed to evaluate the link between in vitro aminoglycoside resistance and different subtypes. We found that *S. flexneri* resistance to these antibiotics was greater than the resistance calculated for *S. sonnei*, and this effect was not influenced by district. In some hospital-based surveillance studies, *S. flexneri* infections have been more frequently detected[25]. One explanation for this could be that *S. flexneri* infections result more frequently in hospitalizations than *S. sonnei* infections. The notion that *S. sonnei* is less virulent than *S. flexneri* is supported by the possibly shorter duration of diarrhea occurring among patients infected with *S. sonnei* than among those with *S. flexneri*. Moreover, the molecular mechanisms of different antimicrobial resistance rate in *Shigella* may be associated with the function of integrons. In *Shigella* species, antimicrobial resistance is often associated with the presence of class 1 and class 2 integrons that contain resistance gene cassettes. Multiple and complex expression regulation mechanisms involving mobile genetic elements in integrons have been developed in the evolution of *Shigella* strains. *S. sonnei* and *S. boydii* strains often contain a single integron of class 2, whereas *S. flexneri* and *S. dysenteriae* strains carry a class 1 integron, either alone or associated with a class 2 integron[26],[27]. Finally, *S. flexneri* could be linked with more complex drug resistant genes. This may be one of the explanations why *S. flexneri* resistance was greater than *S. sonnei*. Information about the rates of *Shigella*-resistance to kanamycin and amikacin in Asian-African regions was not reported, which stresses the need for continuous surveillance of resistance in those countries.

The third significant finding is that, in some countries, the prevalence of *S. sonnei* resistance was greater in 2008-2010. Obviously, the resistance rates among *S. sonnei* have an increasing trend. In some areas, *S. sonnei* has become the primary cause of shigellosis. Some of the *S. sonnei* isolates recovered showed resistance to several kinds of antimicrobial agents. There are distinct phenotypic and genotypic differences in terms of biotypes, antimicrobial susceptibilities and PFGE profiles, and antimicrobial susceptibilities.[70],[72],[73]. An increasing trend in the use of strong antibiotics, such as gentamicin and kanamycin, for shigellosis might be responsible for the acquisition of resistance to these antibiotics in S. sonnei isolates. The increased prevalence of S. sonnei resistance prompted us to suspect that S. sonnei may play a significant role in aminoglycosides−resistance in the future. It is essential to curb the spread of antibiotic resistance and diffusion of S. sonnei should be prevented. Overall, the surveillance of antimicrobial resistance of S. sonnei isolates should be continued, particularly to monitor the emergence of strains fully resistant to aminoglycosides. Thus, analyses of subtypes−based experiences in Shigella resistance can provide an important contribution to the understanding of real−world resistance issues from the perspective of day−to−day medical practice. This analysis also tells us that we should protect against S. flexneri, which caused more hospitalizations in the past, as well as S. sonnei, which caused the majority of shigellosis cases in this study.

Our fourth finding was that, in the pediatric group, the resistance to aminoglycosides was higher than in the adult group population. For children and adults with acute infectious gastroenteritis, the use of specific antimicrobial therapy should be limited to well-defined bacterial and protozoal agents. However, the use of antimicrobial agents in humans for many conditions, including therapy for children with diarrheal disease, is widespread. Although aminoglycosides showed higher in vitro activity against members of the family Enterobacteriaceae, antimicrobial therapy should be considered for specific clinical circumstances including the safety and tolerability of antimicrobial agents, particularly in young children. Considering potential side effects associated with aminoglycoside antibiotics to children, aminoglycoside would not be the best therapeutic choice for gastrointestinal diseases of children. For empiric treatment of diarrheal infections among children, in the report of Abu Elamreen et al.[28],[29], ampicillin and trimethoprim-sulfamethoxazole are most often used. In the meantime, the greater aminoglycoside resistance rates to *Shigella* in the pediatric group cannot be ignored. This meta-analysis provides an important synthesis of the reported aminoglycoside resistance rates for *S. flexneri* and *S. sonnei*. Based on these findings, aminoglycoside resistance is consistently present in a variable proportion of multiple populations. We found that *Shigella* showed greater resistance to gentamicin, kanamycin or amikacin in the pediatric group than it did in the adult group. As interest evolves in the resistance patterns and rates of *Shigella* to aminoglycoside antibiotics, these results can be used to guide treatment decisions and to formulate consensual recommendations for appropriate treatment paradigms, especially for children. The meta-analysis technique used here can help to develop appropriate guidelines governing antibiotic use and to monitor drug resistance trends in different population groups.

Analysis of data on the use of various aminoglycoside antibiotics in different countries and regions of the world indicates that a correlation exists between the selective pressure of antibiotics and the patterns of combinations of aminoglycoside resistance mechanisms. For example, gentamicin has been most frequently used in the USA, while amikacin was used more extensively in Japan. In that time, the significant mechanisms of aminoglycoside resistance in the USA were production of ANT(2″)-I (resistance to gentamicin, tobramycin, dibekacin, and kanamycin), and AAC(3)-I (resistance to gentamicin), whereas in Japan, Europe and Latin America, in addition to ANT(2″)-I, AAC(6′)-I (resistance to amikacin, netilmicin, tobramycin, dibekacin, and kanamycin but not to gentamicin) was identified[20],[30]. The epidemiology of aminoglycoside resistance is becoming more complex, in part because of the multitude of aminoglycoside-modifying enzymes that exist for these antibiotics and also from the presence of disparate additional mechanisms for antibiotic resistance other than enzymatic resistance determinants. Because the genes for the aminoglycoside-modifying enzymes are often located on plasmids or transposons, together with the genes encoding resistance to other classes of antibacterials, the total consumption of non-aminoglycosides can also significantly influence the epidemiological features of aminoglycoside resistance[20].

In summary, this meta-analysis has provided important information on resistance by *S. flexneri* and *S. sonnei* to aminoglycosides in European-American and Asian-African countries. The use of the meta-analysis technique has allowed us to summarize data from individual studies and to determine robust values for both overall resistance and resistance among subgroups. Because identifying resistance patterns can be informative for empiric treatment recommendations, these results will be helpful in developing future guidelines and treatment paradigms for *S. flexneri* and *S. sonnei*, as well as in helping to direct future research on the impact of bacterial resistance and appropriate antimicrobial use.

## Figures and Tables

**Supplementary Table 1:** Studies considered for primary analyses

Author (Reference)	No. of isolates	Location	Children	*Shigella* resistance			*S. flexneri* resistance			*S. sonnei* resistance		
				Gm	Km	Am	Gm	Km	Am	Gm	Km	Am
Sivapalasingam et al. 2006[31]	4432	USA		X	X	X	X	X	X	X	X	X
Wong et al. 2010[22]	616	New York, USA		X			X			X		
Al-Nimri et al. 2009[32]	42	California, USA			X							
Drews et al. 2010[33]	316	Alberta, Canada								X		
Merino et al. 2004[34]	132	Argentina					X			X		
Peirano et al. 2006[35]	306	Brazil		X		X						
Mota et al. 2010[36]	28	Uruguay		X			X			X		
Haukka and Siitonen 2008[37]	228	Finland		X			X			X		
Nógrády et al. 2009[38]	252	Hungary		X	X							
Sire et al. 2008[39]	164	Daker, Senegal		X			X			X		
Putnam et al. 2004[40]	58	Egyptian	X	X		X	X		X	X		X
Mache 2001[14]	77	Ethiopia	X	X	X		X	X		X	X	
Yismaw et al. 2008[41]	214	Ethiopia		X								
Tiruneh 2009[42]	90	Ethiopia		X			X			X		
Opintan and Newman 2007[43]	24	Ghana		X		X						
Djie-Maletz et al. 2008[44]	5	Ghana	X	X		X						
Urio et al. 2001[45]	46	Gaborone, Botswana	X	X			X			X		
Iwalokun et al. 2001[46]	62	Nigeria		X			X			X		
Udo and Eja 2004[47]	123	Calabar, Nigeria	X	X								
Davies and Karstaedt 2008[48]	34	Soweto		X		X						
Temu et al. 2007[49]	69	Mwanza,Tanzania		X			X					
Moyo et al. 2011[50]	15	Tanzania	X	X			X					
Samie et al. 2009[51]	51	Vhembe		X								
Yu et al. 2007[15]	459	China		X								
Zhu et al. 2011[52]	58	Henan		X			X					
Ding et al. 2005[53]	290	Shanghai, China		X			X			X		
Zhu et al. 2009[54]	215	Shanghai, China				X			X			X
Gu et al. 2008[27]	26	Nanjing, China		X		X						
Wang et al. 2005[55]	331	Hebei, China		X			X			X		
Xia et al. 2011[19]	87	Henan, China					X					
Jones et al. 2004[56]	90	Amazn river basin		X								
Navaneeth et al. 2001[57]	12	Bangalore, India		X			X			X		
Rahman et al. 2007[58]	266	Bangalore, India		X			X			X		
Bercion et al. 2008[59]	47	Bangui		X								
Ashkenazi et al. 2003[60]	617	Israel		X			X			X		
Niyogi et al. 2001[61]	34	India	X	X			X			X		
Dutta et al. 2002[62]	101	India		X		X						
Taneja et al. 2004[63]	54	India		X		X	X		X	X		X
Uppal et al. 2004[64]	56	Delhi, India	X	X			X			X		
Mamatha et al. 2007[65]	68	India		X								
Nandy et al. 2010[66]	516	Kolkata, India		X		X						
MoezArdalan et al. 2003[67]	98	Tehran, Iran		X	X	X	X	X	X	X	X	X
Nowroozi et al. 2006[68]	142	Iran	X				X					
Farshad et al. 2006[69]	82	Iran	X	X		X	X		X	X		X
Ranjbar et al. 2009[70]	200	Iran			X	X						
Hamedi 2009[71]	98	Iran	X	X		X						
Ashtiani et al. 2009[72]	1571	Tehran, Iran		X	X	X						
Pourakbari et al. 2010[73]	682	Tehran, Iran	X	X	X	X						
Soltan Dallal et al. 2011[74]	84	Tehran, Iran						X	X			
Izumiya et al. 2009[75]	195	Japan								X	X	
Hirose et al. 2005[76]	58	Japan									X	
Ahmed et al. 2006[77]	21	Hiroshima, Japan			X			X			X	
Oh et al. 2003[78]	67	Korea									X	
Seol et al. 2006[79]	110	Korea								X	X	
Jin et al. 2010[80]	66	Seoul, Korea								X		
Jamal et al. 2010[81]	142	Kuwait& Arab		X		X						
Wilson et al. 2006[19]	66	Pokhara, Nepal		X			X			X		
Abu Elamreen et al. 2007[82]	6	Gaza,Palestine	X	X		X						
Abu Elamreen et al. 2008[28]	28	Gaza,Palestine	X	X		X						
Chompook et al. 2005[25]	144	Saraburi,Thailand		X	X	X	X	X	X	X	X	X
Banajeh et al. 2001[83]	25	Yemen	X	X								
Al-Moyed et al. 2006[84]	105	Saraburi, Yemen		X								
Meng et al. 2011[85]	41	Cambodia	X	X								
Debdutta Bhattacharya, et al. 2012[86]	33	Andaman Islands, India		X		X						
Zhang W et al. 2011[87]	230	china		X			X			X		
Shiferaw B, et al. 2012[88]	1118	USA		X	X		X	X		X	X	
Shamsizadeh A, et al. 2012[89]	154	Iran	X	X								
Tajbakhsh M, et al. 2012[90]	37	Tehran,Iran		X			X			X		

Gm: Gentamicin; Km: Kanamycin; Am: Amikacin.
